# A Heterozygous Variant in the GABBR2 Gene in a Girl With Clinical Classic Rett Syndrome

**DOI:** 10.1002/mgg3.70303

**Published:** 2026-09-27

**Authors:** Jenny Klintenstedt, Peter Baeck, Ingegerd Witt Engerström, Cecilia Gunnarsson

**Affiliations:** ^1^ Department of Clinical Genetics, and Department of Biomedical and Clinical Sciences Linköping University Linköping Sweden; ^2^ Department of Pediatrics Kalmar County Hospital Kalmar Sweden; ^3^ National Swedish Rett Center Jämtland County Council Östersund Sweden; ^4^ Center for Rare Diseases in Southeast Region of Sweden Linköping University Linköping Sweden

**Keywords:** *GABBR2*, genotype–phenotype correlation, MECP2‐negative, neurodevelopmental disorder, Rett syndrome

## Abstract

**Background:**

Rett syndrome (RTT) is a neurodevelopmental disorder mainly affecting females and may start with seemingly normal early development but leads to developmental stagnation, regression, and characteristic neurological symptoms. While most cases involve *MECP2* variants, other genes have been implicated in RTT and RTT‐like phenotypes, but the underlying molecular mechanisms remain incompletely understood.

**Methods:**

We describe a girl fulfilling the clinical diagnostic criteria for classic RTT in whom standard genetic testing, including *MECP2* and *CDKL5*, was normal. Trio‐based whole‐exome sequencing was used to identify an alternative genetic cause that was confirmed using Sanger sequencing.

**Results:**

A heterozygous *de novo* variant in *GABBR2* (NM_005458.7:c.1699G>A; p.Ala567Thr) was identified as the only clinically relevant genetic finding. The patient fulfilled the clinical diagnostic criteria for classic RTT and exhibited developmental stagnation, progressive impairment of purposeful hand use, characteristic stereotypic movements, autonomic dysfunction, and behavioral disturbances.

**Conclusion:**

This patient expands the phenotypic spectrum associated with *GABBR2* variants and, together with previously reported cases, provides further evidence that *GABBR2*‐related disease may present with a clinical RTT phenotype. These findings support a role for *GABBR2*‐mediated GABAergic signaling in the pathophysiology of RTT and highlight the importance of considering *GABBR2* in the genetic evaluation of *MECP2*‐negative patients with a clinical RTT phenotype.

## Graphical abstract

A heterozygous de novo *GABBR2* variant was identified in a girl with clinical classical Rett syndrome. Comparison with previously reported cases suggests that *GABBR2* variants should be considered in the genetic evaluation of individuals with *MECP2*‐negative Rett syndrome.

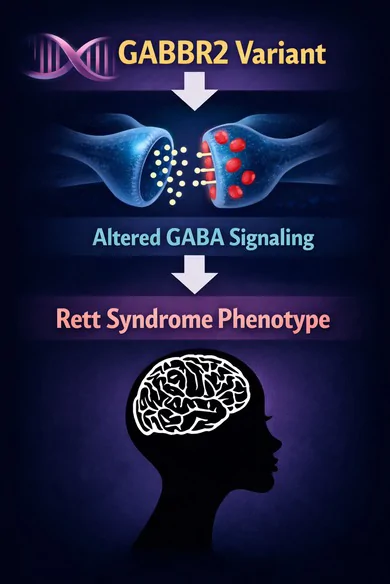

## 1 Introduction

Rett syndrome (RTT; OMIM #312750) is a neurodevelopmental disorder almost exclusively affecting girls. Pathogenic variants in the *MECP2* gene, located on the X chromosome, are the major cause of RTT (Halbach et al. 2016). *MECP2* encodes a methyl‐DNA‐binding protein, MeCP2, which is an important epigenetic regulator involved in neuronal gene regulation, functioning both as a transcriptional repressor and activator (Liu et al. 2025).

Affected infants may appear normal at birth and in early motor development but often show subtle early signs, such as mild hypotonia, in retrospect. Gross motor development is gradually delayed, followed by general developmental stagnation and subsequent regression with loss of or setbacks in previously acquired skills including purposeful hand use and communication. Slower growth in head circumference, body height, and weight is common. Hand‐mouth stereotypies, bruxism, intense crying spells, constipation, sleep and feeding difficulties may gradually emerge (Witt Engerström 1987). Many patients enter a relatively stable plateau phase with some improved functions, such as better communication and eye contact. Screaming spells diminish, but dystonia and scoliosis can develop. Autonomic imbalance with cardiorespiratory symptoms, respiratory dysrhythmia and epilepsy may develop (Julu et al. 2008). A recent natural history study in individuals with RTT demonstrated that most developmental skill acquisition occurs before 6 years of age, that skill regain, when it occurs, generally also occurs before this age, and that skill loss most often occurs within 2 years of acquiring that skill (Neul et al. 2026). Although the sequence of clinical signs is relatively characteristic, their timing and severity vary between individuals (Kerr and Witt Engerström 2001).

RTT is classified as classic/typical or atypical according to established diagnostic criteria. In the classic group, a *MECP2* variant is found in 95%–97% of patients; in the atypical group, 50%–70% (Neul et al. 2010). Cyclin‐dependent kinase‐like 5 (*CDKL5*) and Forkhead box protein G1 (*FOXG1*) variants were previously described as causes of atypical RTT (Neul et al. 2010) but are now recognized as distinct disorders. This study used whole exome sequencing (WES) to identify the disease‐causing genetic variant in a girl with a clinical diagnosis of classic RTT but lacking a pathogenic *MECP2* variant. In addition to describing the clinical and genetic findings, we compare phenotype with previously reported cases to explore the genotype‐phenotype correlations.

## 2 Methods

### 2.1 Editorial Policies and Ethical Considerations

Approved by the regional ethical review board in Linköping (Decision number 2013/364‐31). Informed consent for this report has been obtained from the patient's family.

We performed conventional cytogenetic analysis on GTG‐banded chromosomes from cultured lymphocytes in the clinical genetics laboratory in Linköping according to standard techniques. The analysis revealed a 46,XX, normal female karyotype at the 550 band level.

The Linköping cytogenetics laboratory performed a microarray hybridization analysis on DNA extracted from peripheral blood using Affymetrix Human Mapping 250 K SNP Array technology (Affymetrix, Santa Clara, CA; www.affymetrix.com). This platform can genotype average 262,000 SNPs distributed across all autosomes and the X‐chromosome. Results showed normal copy variation; arr(1‐22,X)x2.

Given that the clinical picture suggested RTT or a differential diagnosis, the coding regions of *MECP2* and *CDKL5* were analyzed by DNA sequencing using DNA extracted from peripheral blood, yielding normal results. MLPA (multiplex ligation‐dependent probe amplification) analysis of *MECP2* and *CDKL5* detected no deletions or duplications.

WES was performed as a trio (patient and parents). Library preparation and bioinformatic analysis were performed by Oxford Gene Technology (OGT, Begbroke, UK). Briefly, paired end sequencing libraries captured from exonic sequences were prepared using Agilent's Sure Select Protocol Version 1.2 according to the manufacturer's instruction (Agilent, Santa Clara, US). Paired‐end sequencing libraries were performed on an Illumina HiSeq 2000 instrument using TruSeq v3 chemistry. Reads were mapped to the hg19/b37 reference genome using Burrows–Wheeler Aligner (version 0.6.1), and variants were called using Unified Genotyper from the Genome Analysis Toolkit (GATK version 1.6). Local realignment was performed using GATK, and duplicate reads were marked using Picard (version 1.62). Base quality scores were recalibrated using covariance recalibration (GATK) before variant calling. Variants were annotated using dbSNP release 135 and Ensembl. In total, 12.45 gigabases of sequence data were read and aligned at high quality, with a minimum of 83.36% of target regions covered at 20X depth. Variants were filtered for *de novo* inheritance and further filtered by selecting those found in genes associated with clinical symptoms in the OMIM database. Non‐relevant phenotype associations in OMIM were discarded. Further analysis removed likely sequencing artifacts and was done to predict the effect of the variant. Variants that did not introduce a new amino acid, affect splicing, cause a frameshift, or gain/loss of stop codon in a coding part of a gene were excluded. Suspected variant was confirmed using Sanger sequencing performed at the Department of Clinical Genetics, Uppsala, Sweden.

## 3 Results

### 3.1 Clinical Description

The girl was born in 2009 as the first child to non‐consanguineous Swedish parents. The pregnancy was uneventful, with breech delivery at week 38+3. Birthweight was 2760 g, height 47 cm, head circumference 33 cm and Apgar score 7‐8‐9. She was initially slightly hypotonic with feeding problems for a few days. Her development in the first months appeared normal.

#### 3.1.1 6 Months

Babbling and smiling but hypotonic with poor trunk stability, delayed motor development (did not grasp, kept her hands clasped, seldom turns from back to stomach, was late to hold head independently) and feeding difficulties. Referred to the pediatric department for a thorough medical investigation: chromosome analysis, metabolic testing, muscle biopsy, and a mitochondrial workup: all inconclusive.

#### 3.1.2 11 Months

Communicated with sounds and smiles but lacked direct eye contact. Unstable trunk, could not sit unsupported. Writhing hand movements midline, still unable to grasp purposefully, and frequent screaming spells. Dependent on feeding tube. Her parents reported decreased pain sensitivity.

#### 3.1.3 12 Months

Percutaneous Endoscopic Gastrostomy inserted due to lack of interest in eating. Fed exclusively with PEG. Genetic testing for RTT (*MECP2*) and chromosomal microarray analysis/SNP array were normal. Brain MRI suggested some degree of white matter loss, most pronounced bilaterally in the fronto‐parietal region, and parieto‐occipitally on the left side.

#### 3.1.4 14 Months

Severe screaming periods, dystonia in both feet. Developed self‐injurious hand biting.

#### 3.1.5 16 Months

Did not bear weight through her feet but played with her feet using he hands. Still not sitting independently. Disturbed sleep. No eye contact but could grasp and transfer objects between hands.

#### 3.1.6 2 Years

Could stand in a standing shell, still no independent sitting. Tooth grinding (bruxism) and prolonged screaming episodes lasting hours. Said a few sounds but with no clear intent. Some eye contact and was reluctant to grasp objects.

#### 3.1.7 Two and a Half Years

Slept well for 10–12 h. No real use of hands. Autism spectrum disorder was suspected.

#### 3.1.8 3 Years

Still non‐ambulant, no language or eye contact. She exhibited self‐biting and self‐hitting behaviour, and was diagnosed with autism.

#### 3.1.9 7 Years

Follow‐up MRI was normal. Following evaluation by an RTT specialist, she was considered to fulfill the diagnostic criteria for classic RTT, including partial loss of acquired purposeful hand function, loss of acquired spoken language, absent ability to walk, and stereotypic hand movements. Could sit unsupported (long sitting), communicate with smiles and eye gaze, and return a rolling ball. Respiratory dysfunction with periods of intense hyperventilation and breath‐holding. Bruxism and stereotypic hand‐mouth movements.

#### 3.1.10 Summary of Developmental Course

Development appeared normal during the first months of life, but abnormalities became evident by 6 months of age with hypotonia, feeding difficulties and delayed motor milestone development. During the second year of life, developmental stagnation was accompanied by progressive impairment of purposeful hand use and reduced social communication, while characteristic RTT features including hand stereotypies, bruxism, respiratory dysfunction, and self‐injurious behavior gradually emerged. Later in childhood she showed partial improvement in eye‐mediated communication and sitting ability, whereas severe motor impairment and absent spoken language persisted.

### 3.2 Genetic Finding

Trio‐based whole‐exome sequencing identified a heterozygous de novo variant in *GABBR2* (chr9:101133817; NM_005458.7: c.1699G>A; p.Ala567Thr). This was the only clinically relevant de novo variant and was confirmed by Sanger sequencing.

### 3.3 Clinical Follow Up

At the last clinical follow‐up, the 15‐year‐old girl predominantly struggled with behavioral problems, including screaming spells, weak and complicated interpersonal contact and self‐injury. Her behavioral problems have been lifelong but have progressively worsened over time, showing a cyclicity pattern: 2 weeks of better behavior, attending special school 4–5 days a week, followed by 2 weeks of increased agitation, screaming and self‐injury, attending school only 2 days a week. Her self‐injurious behavior appeared to be voluntary and stress‐related; however, in the absence of other purposeful voluntary movements this was difficult to assess. Menarche at 14 years of age, with no clear behavioral pattern related to the menstrual cycle. Various medications have been tried to improve behavior and sleep, including clonidine, benzodiazepines, aripiprazole, sertraline, melatonin, promethazine, and risperidone. Risperidone had the best effect but its effect only lasted a few hours. Benzodiazepines, interestingly, had no effect on self‐injurious behavior.

## 4 Discussion

Our patient was clinically diagnosed with classic RTT following assessment by an RTT specialist according to the 2010 criteria (Neul et al. 2010). Basic genetic investigations, including *MECP2* and *CDKL5* sequencing, were normal. SNP microarray analysis excluded microdeletions and microduplications as a likely cause. However, subsequent WES identified a pathogenic variant in *GABBR2* c.1699 G>A (p.Ala567Thr). Comparison with previously reported cases supports an association between *GABBR2* and an RTT phenotype.

### 4.1 *GABBR2* and Its Function

The *GABBR2* gene, located at cytoband 9q22.33, codes for a membrane protein in the G protein‐coupled receptor family (Chao et al. 2010). The protein, consisting of 941 amino acids, forms seven transmembrane domains (TMs). The phenotype depends on the variant's site and TM involved (D'Onofrio et al. 2022). γ‐aminobutyric acid (GABA), the main inhibitory neurotransmitter of the CNS, activates two major receptor classes: GABA_A_ and GABA_C_ receptors, which function as chloride channels, and GABA_B_ receptors, which are metabotropic receptors. *GABBR2* encodes a subunit of the GABA_B_ receptor, which inhibits neuronal activity by regulating neurotransmitter release (Feng et al. 2022). It is highly expressed in various brain regions (Feng et al. 2022). Chao et al. (2010) showed that GABAergic dysfunction is critical in RTT phenotypes. Mice with *MECP2* “deficiency in GABAergic neurons initially exhibit normal behavior, then develop forepaw stereotyped movements, compulsive grooming, increased sociability, impaired motor coordination, learning/memory deficits, abnormal EEG hyperexcitability, severe respiratory dysrhythmias, and premature lethality” (page 6) (Chao et al. 2010).

*MECP2* dysfunction has been associated with impaired GABAergic signaling and imbalance between neuronal excitation and inhibition, mechanisms implicated in RTT pathophysiology. MeCP2 is essential for the normal function of GABAergic neurons and even subtle dysfunction of these neurons contributes to numerous neuropsychiatric phenotypes such as RTT (Chao et al. 2010). Since *GABBR2* encodes a subunit of the GABA_B_ receptor involved in inhibitory neurotransmission, pathogenic variants may affect overlapping GABAergic pathways, thereby contributing to an RTT spectrum phenotype.

### 4.2 Published Patients

Lopes et al. (2016) described a girl with developmental stagnation at 7 months, followed by regression and severe intellectual disability. She was classified as having an RTT variant due to her absence of language, hand stereotypies and lack of hand use, hyperventilation, bruxism, abnormal sleep cycle, crying spells, autistic features, eye pointing, and small feet. She never had seizures. They found the same *GABBR2* (p.Ala567Thr) variant as in our patient, also *de novo*.

The same variant was reported in a patient clinically meeting the criteria for RTT, but without epilepsy (Lucariello et al. 2016). Notably, this patient also has a variant in another gene (*ATP8B1*).

Yoo et al. (2017) screened 34 *MECP2*‐negative patients meeting RTT clinical criteria by WES, detecting the exact same *GABBR2* (p.Ala567Thr) variant in two patients. They provided functional evidence for reduced receptor function of this variant using in vitro and in vivo models.

Seventeen heterozygous de novo *GABBR2* variants were identified in a literature review in patients with ID, ASD, and drug‐resistant epilepsy. The variant *GABBR2* (p.Ala567Thr) affects a highly conserved residue within the TM3 domain, with 7 of 17 patients having this exact variant (D'Onofrio et al. 2022).

A literature search now identifies a total of 10 patients with the *GABBR2* (p.Ala567Thr) variant, making our patient the eleventh. The most commonly reported characteristics include ID, ASD, and an RTT‐like phenotype. Only about half have epilepsy, despite connections between *GABBR2* and epileptic encephalopathy (EE). The clinical characteristics of previously reported patients carrying the *GABBR2* (p.Ala567Thr) variant are presented in Table 1.

**TABLE 1:** Clinical characteristics of previously reported patients with the *GABBR2* (p.Ala567Thr) variant.

Author (year)	Age, sex	ID	Epilepsy	Hypotonia	ASD	RTT phenotype	Behavioral issues^a^
Lopes et al. (2016)	19, F	Y	N	Y	Y	Y, atypical	Y (no self‐injury)
Lucariello et al. (2016)	2, —	—	N	—	—	Y, —	—
Deciphering Developmental Disorders Study (2017)	—, M	Y	—	—	—	—	—
Deciphering Developmental Disorders Study (2017)	—, M	Y	—	—	—	—	—
Yoo et al. (2017)	9, M	—	Y	—	—	Y, atypical	Y (no self‐injury)
Yoo et al. (2017)	14, F	—	Y	—	—	Y, atypical	Y (no self‐injury)
Yoo et al. (2017)	28, M	Y	N	—	Y	Y, atypical	Y (no screaming spells)
Yoo et al. (2017)	16, M	—	N	—	Y	Y, atypical	—
Carneiro et al. (2018)	11, M	Y	Y	—	—	—	—
Takata et al. (2018)^b^	14, F	Y	Y	Y	Y	—	Y (no screaming spells)
Our patient	15, F	Y	N	Y	Y	Y, classic	Y

Abbreviations: ASD, autism spectrum disorder; F, female; ID, intellectual disability; M, male; N, no; Y, yes.

^a^ Such as screaming spells, sleep disturbance, and self‐injury.

^b^ Information was obtained through direct contact with the corresponding author, Dr. Matsumoto, M.D., Ph.D. and is not included in the published paper.

Of these patients, 7 of 11 have clinical RTT. This highlights the importance of the GABAergic pathway as a possible explanation for clinical RTT features. Our results suggest that *GABBR2*‐mediated GABA signaling is an important factor influencing disease severity.

### 4.3 What Determines the Phenotype?

There are two phenotype entries of the *GABBR2* gene in OMIM (#617904 and #617903), not yet formally linked to RTT. One of these, Neurodevelopmental disorder with poor language and loss of hand skills (NDPLHS), is, however, described as having a phenotype reminiscent of RTT. Both our patient and other published patients show a substantial overlap between these entries, particularly in neurodevelopmental impairment, behavioral features, and variable epilepsy, rather than clustering exclusively within one. This supports the interpretation that GABBR2‐related disease may represent a phenotypic spectrum, with our patient exhibiting one of the more RTT‐like phenotypes reported to date.

Yoo et al. (2017) suggest that “*GABBR2* may lie at the intersection of RTT‐ or EE causing pathways and may have a role in differentiating the two diseases despite the observation that the two diseases display distinct enrichment of biological pathways” (page 472). They propose that receptor activity level may determine the phenotype.

Similarly, D'Onofrio et al. (2022) suggest that the phenotype depends on the variant's location and TM domain involved. The proposed disease mechanism involves underactivation at the neuromuscular junction and/or in spinal motor control centers. They state: “pathogenic mechanisms may be multiple involving other genes through neuronal circuits” (page 472).

### 4.4 Treatment

Our patient's lack of response to benzodiazepines may be due to the variant affecting the GABA receptor, since benzodiazepines enhance GABAergic inhibition through the GABA_A_ receptor, while this variant affects the GABA_B_ receptor (Bounds and Patel 2024). Baclofen, an agonist for the GABA_B_ receptor, used for muscle relaxation, is the only drug available acting on this receptor. Limitations for use in this setting are low penetration of the blood‐brain barrier, short duration of action, and rapid tolerance development (de Leon and Tadi 2023).

The GABA_B_ receptor also seems involved in pain signals, stress, anxiety, and affective disorders. More research is needed, but potential treatments include transcranial magnetic stimulation (partly mediated by GABA_B_ receptor‐mediated inhibition) and more selective drugs (mainly Positive/Negative Allosteric Modulators) affecting the GABA_B_ receptor with fewer side effects than baclofen (Rose and Wickman 2022). Manipulating the receptor could significantly benefit patients with this variant, which reduces receptor activity to approximately 30% of wild‐type activity (Yoo et al. 2017). Connections between this receptor, neurodegeneration, and epilepsy are also noted by Rose and Wickman (2022).

## 5 Conclusion

Patients with an RTT phenotype may have pathogenic variants in genes other than *MECP2*. As the genetic testing strategy increasingly shifts toward broader sequencing approaches, the recurrent *GABBR2* (p.Ala567Thr) variant may prove to be more common than currently recognized. Broad sequencing approaches, such as whole genome sequencing, combined with phenotype‐driven variant prioritization may reduce the risk of missing variants in genes such as *GABBR2*. However, since cost and access still influence diagnostic strategies, we believe *GABBR2* should be included if a less comprehensive testing approach is chosen. Given that this specific *GABBR2* variant appears to be recurrent, *GABBR2* should be considered a candidate gene for testing in patients with RTT spectrum phenotypes. Given the severe and lifelong neurodevelopmental burden associated with RTT spectrum phenotypes, future therapies targeting GABAergic dysfunction may make early molecular diagnosis increasingly important.

## Author Contributions

**Peter Baeck:** investigation, writing – review and editing. **Cecilia Gunnarsson:** conceptualization, project administration, supervision, writing – review and editing, investigation. **Jenny Klintenstedt:** conceptualization, investigation, visualization, writing – original draft, writing – review and editing. **Ingegerd Witt Engerström:** investigation, writing – review and editing.

## Funding

This work was supported by the Medical Research Council of Southeast Sweden (Forskningsrådet i Sydöstra Sverige; grant #1012769). The funders had no role in the study design, data collection, analysis, and decision to publish or in the preparation of the manuscript.

## Conflicts of Interest

The authors declare no conflicts of interest.

## Data Availability

The data that support the findings of this study are available on request from the corresponding author. The data are not publicly available due to privacy or ethical restrictions.
